# Clinical characteristics and gene analysis of long QT syndrome in 15 children

**DOI:** 10.3389/fped.2025.1571495

**Published:** 2025-04-17

**Authors:** Li Yiwei, Gong Peiwen, Lv Tiewei, Sun Huichao

**Affiliations:** Department of Cardiology, National Clinical Research Center for Child Health and Disorders, Ministry of Education Key Laboratory of Child Development and Disorders, Key Laboratory of Children's Vital Organ Development and Diseases of Chongqing Health Commission, National Clinical Key Cardiovascular Specialty, Children's Hospital of Chongqing Medical University, Chongqing, China

**Keywords:** long QT syndrome, children, clinical characteristics, gene mutation, *KCNQ1*

## Abstract

**Objective:**

To elucidate the genetic and clinical characteristics of children diagnosed with long QT syndrome (LQTS) at our institution.

**Study design:**

This was a retrospective study. Clinical data and gene detection results of 15 cases diagnosed with congenital LQTS at our center from January 1, 2018 to December 30, 2023 were collected and analyzed using independent sample *t*-test and Levene's test for equality of variances.

**Results:**

The 15 LQTS cases included 7 females and 8 males. The mean age of onset for females (11.83 ± 3.48 years) was later than that for males (8.06 ± 2.50 years), and the mean QTc value for females (564.57 ± 20.72 ms) was higher than that for males (502. 25 ± 48.62 ms), both differences were statistically significant (*P* < 0.05). Intense exercise and psychological stress are the most common predisposing factors in these cases. Gene mutations were found in 14 of the 15 cases and most mutations (13/14) were inherited from parents. According to the mutation sites, the most common mutation type was missense mutations (11/15). One genetically exclusive case with a Schwartz score of 4 was clinically diagnosed with LQTS after excluding other diseases.

**Conclusions:**

In this cohort, age of onset and QTc value are different between male and female. The most common primary symptom of LQTS is syncope. Most LQTS patients have a mutated gene inherited from their parents, and the most common pathogenic gene is *KCNQ1*.

## 1 Introduction

Among non-ischemic aborted cardiac arrest (ACA) survivors with genetic heart disease, 26.7% exhibit myocardial ion channel diseases. Among these pediatric patients, long QT syndrome (LQTS) is the main cause of their suffering (1). LQTS is an ion channelopathy characterized by cardiac adverse events such as recurrent syncope, ACA and sudden cardiac death. On the electrocardiogram, it can be manifested including prolonged QTc (typically >460 ms), ventricular tachycardia, particularly torsades de pointes, T-wave abnormalities (2–4). Most LQTS patients carry gene mutations that incorrectly encode ion channels proteins involved in myocardial electrophysiological activity. This results in changes in ionic currents due to structural abnormalities at different stages of the action potential of cardiomyocytes, leading to delayed repolarization of cardiomyocyte (2, 5).

At present, a total of 17 genes are reported to be associated with LQTS, among which the pathogenicity of *KCNQ1*, *KCNH2* and *SCN5A* is definitive (6). Genetic epidemiological surveys based on those three genes show that the prevalence of LQTS ranges from 1/1,000 to 1/2,400 (7–10). Pathogenic or likely pathogenic mutations could be identified in 2.7% of sudden cardiac death (11), and as a genetically related disease, 7% of the first- and second- degree family members of individuals who suffered from sudden unexplained death or ACA could be confirmed or suspected as LQTS (12, 13). However, in clinical practice, there may be incomplete concordance between genotype and phenotype. Studies have shown that approximately 44%–73% of the mutation carriers are asymptomatic at hospital visits (14–17), and around 11%–28% of the LQTS patients presenting with syncope have no clear pathogenic gene mutations (18, 19). Timely diagnosis and treatment are critical.

Therefore, this article aims to present the clinical features and gene mutations of 15 children suspected of LQTS based on their clinical symptoms, and analyze the characteristics to advance the understanding of gene mutation sites, diagnosis and differential diagnosis of LQTS.

## 2 Methods

### 2.1 Study subjects

Children diagnosed with Long QT Syndrome (LQTS) who were hospitalized in the Department of Cardiology at the Children's Hospital of Chongqing Medical University between January 1, 2018, and December 30, 2023, were enrolled. Clinical data were retrospectively collected and analyzed. Patients with acquired LQTS or concurrent hereditary syndromes were excluded. Ethical approval was granted by the Ethics Committee of Children's Hospital of Chongqing Medical University [Approval number: Ethics Review (Clinical Research) No. 456, 2024]. Written informed consent was obtained from both participants and their legal guardians for genetic testing and data utilization.

### 2.2 Diagnostic criteria of LQTS

The diagnostic criteria for LQTS include: (1) Schwartz score of >3 points based on electrocardiogram, clinical symptoms, family history, and genetic testing results; (2) QTc ≥480 ms; (3) QTc ≥460 ms with syncope, with the lower limit of QTc in HRS/EHRA/APHRS expert consensus (2013) being 20 ms higher. Secondary causes were rigorously excluded prior to diagnosis (3, 4, 20).

### 2.3 Data collection

Demographic and clinical parameters included gender, age, precipitating factors, prodromal symptoms, main symptoms, QTc values, Schwartz scores, and family history. The genetic whole-exome testing of all the patients was performed by Beijing MyGenostics Gene Technology Co., Ltd. The mutation genes and mutation sites associated with clinical phenotypes were analyzed by American College of Medical Genetics and Genomics (ACMG) guidelines.

### 2.4 Structural modeling

FASTA format of amino acid sequence of KCNQ1 was searched on UniProt (https://www.uniprot.org/, ID: 51787-1) and the structural model was made on SWISS MODEL (https://swissmodel.expasy.org/): the template was Potassium voltage-gated channel subfamily KQT member 1, sequence coverage was 100%, global model quality estimation was 0.68.

### 2.5 Statistical analysis

Data utilized SPSS 25.0. Demographic data were reported as mean ± SD, median (range), number, percentage. Group comparisons employed independent samples *t*-test and Levene's test for equality of variances. *P* < 0.05 was considered statistically significant.

## 3 Results

### 3.1 General observations

A total of 15 pediatric LQTS cases were met the inclusion criteria (Table 1). The age at diagnosis ranged from 3 years to 15 years, with a median age was 9.9 years. The cohort comprised 8 males (53.3%) and 7 females (46.7%). The mean age at diagnosis was 11.83 ± 3.38 years for females and 8.06 ± 2.50 years for males, with a significant difference between them (*P* = 0.030) (Table 2).

**Table 1:** Basic clinical information of the 15 cases.

Proband	Gender	Age	Predisposing factor	Presence or absence of prodromal symptoms	Main symptom	Schwartz score before gene testing	QTc at admission (ms)	Treatment
P1	M	9 years and 11 months	Intensive exercise	A	Vertigo	3	486	β-blocker
P2	F	14 years and 3 months	Psychological stress	A	Amaurosis	5	566	β-blocker
P3	M	7 years and 2 months	—	A	Syncope	1	423	β-blocker
P4	M	10 years	—	A	Syncope	4	557	β-blocker
P5	F	6 years and 4 months	Psychological stress fatigue	P	Syncope	6	545	β-blocker
P6	F	11 years	Psychological stress Intensive exercise High ambient temperature	A	Respiratory and cardiac arrest	6	588	β-blocker
P7	M	3 years and 11 months	Psychological stress Intensive exercise	P	Syncope	5	563	β-blocker
P8	M	12 years and 1 month	—	P	Precordial pain	3	541	β-blocker
P9	M	7 years and 4 months	Intensive exercise	P	Syncope	5	486	β-blocker
P10	M	7 years	Intensive exercise	P	Syncope	5	463	β-blocker
P11	F	14 years and 6 months	Psychological stress	P	Syncope	5	578	β-blocker
P12	F	13 years	Psychological stress Intensive exercise	A	Convulsion	3	588	β-blocker
P13	F	15 years and 7 months	Intensive exercise	P	Syncope	6	547	β-blocker
P14	M	7 years and 1 month	Intensive exercise	P	Syncope	6	499	β-blocker
P15	F	8 years and 2 months	—	P	Syncope	4	540	β-blocker

“F” means “female”, “M” means “male”. “A” means “absence”, “P” means “Presence”.

**Table 2:** The schwartz score and QTc value.

Test variable	Group	Number of cases	Average value ± standard deviation	Levene test for equality of variances	*P* value of *t*-test
Age(years)	Female	7	11.83 ± 3.48	0.264	0.030
	Male	8	8.06 ± 2.50		
Schwartz score	Female	7	5.00 ± 1.15	0.361	0.195
	Male	8	4.00 ± 1.60		
QTc (ms)	Female	7	564.57 ± 20.72	0.061	0.008
	Male	8	502.25 ± 48.62		
QTc (ms)	Syncope*	11	526.27 ± 51.75	0.485	0.527
	Non-syncope	4	545.25 ± 43.92		
Schwartz score	Syncope	11	4.82 ± 1.47	0.668	0.125
	Non-syncope	4	3.50 ± 1.00		

Syncope*: including syncope, respiratory and cardiac arrest cases.

**Figure 1:**
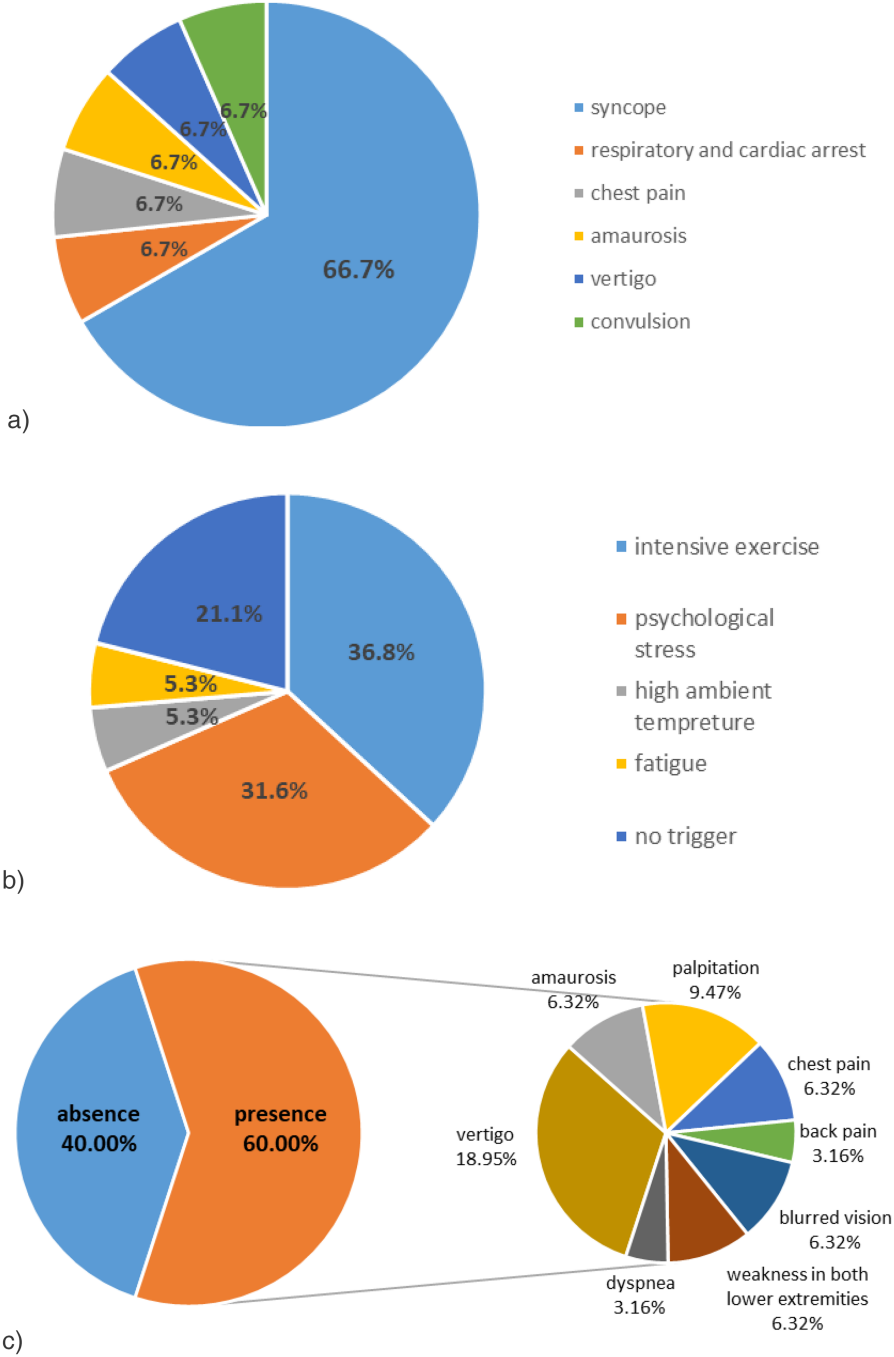
Clinical features of the 15 cases. **(a)** Syncope is the most common primary clinical manifestation in these cases. **(b)** Intense exercise and psychological stress are the most common predisposing factors in these cases. **(c)** Various prodromal symptoms of the cases.

Primary clinical manifestations of the cases included: syncope (10/15, 66.7%), respiratory and cardiac arrest (1/15, 6.7%), precordial pain (1/15, 6.7%), amaurosis (1/15, 6.7%), convulsion (1/15, 6.7%) and vertigo (1/15, 6.7%) (Figure 1a). Identifiable predisposing factors were reported in symptomatic cases: intense exercise (7/19, 36.8%), psychological stress (6/19, 31.6%), high ambient temperature (1/19, 5.3%), and fatigue (1/19, 5.3%) (Figure 1b). Four cases lacked identifiable triggers.

Nine patients (40.0%) presented with prodromal symptoms including: vertigo (6/19, 31.6%), palpitations (3/19, 15.8%), amaurosis (2/19, 10.5%), chest pain (2/19, 10.5%), blurred vision (2/19, 10.5%), bilateral lower limb weakness (2/19, 10.5%), dyspnea (1/19, 5.3%), and back pain (1/19, 5.3%). Co-occurrence of multiple prodromal symptoms was observed (Figure 1c).

### 3.2 Schwartz score and QTc

Schwartz score across the cohort ranged from 1 to 6 (mean 4.5 ± 1.5). No significant sex-based differences were observed in Schwartz scores (females: 5.00 ± 1.16 vs. males: 4.00 ± 1.60; *P* = 0.195). QTc intervals ranged from 423 ms to 588 ms (mean 531 ± 49 ms). Females demonstrated significantly prolonged QTc values compared to males (564.57 ± 20.72 ms vs. 502.25 ± 48.62 ms; *P* = 0.008). According to syncope status, QTc intervals showed no significant difference between syncope (526.27 ± 51.75 ms) and none-syncope groups (545.25 ± 43.92 ms; *P* = 0.527), and Schwartz scores similarly demonstrated no statistically significant differences (syncope: 4.82 ± 1.47 vs. none-syncope: 3.50 ± 1.00; *P* = 0.125) (Table 2).

### 3.3 Family history

Maternal lineages exhibited distinct pathological patterns: hearing impairment in P1's mother, QTc prolongation in mothers of P2, P4, P9, and P15, with P4's mother showing suspected epilepsy and arrhythmogenic syncope. The sibling pair P5-P6 manifested recurrent syncope (P5) and sudden cardiac arrest (P6). Multigenerational involvement was observed in P9-P15, including paternal syncope (P11) and QTc prolongation (P15), alongside transgenerational neurological events in P10's grandmother. Affected siblings P13-P14 showed maternal syncopal episodes, while P12's mother experienced tachycardia-induced syncope. Notably, P7 and P8 presented no inheritable cardiovascular pathology.

### 3.4 Gene features

Pathogenic or likely pathogenic 14 variants were detected in 12 cases which were highly related to the clinical phenotype: *KCNQ1* (7), *KCNE1* (1), *KCNH2* (2), *KCNJ2* (1), *CACNA1C* (1). In P4 and P12, two variants of *KCNH2* were found, but the significance of the mutations cite was uncertain according to ACMG. No related mutant gene was detected in P15. Except for the spontaneous mutation of *CACNA1C (c.1216G>A)* in P8, all mutations were parental origin. According to the mutation sites, there were 11 missense mutations, 3 deletion mutations and 1 duplication mutation (Table 3).

**Table 3:** Gene results and family history of the cases.

Patient	Gene	Variant	Classification of ACMG	Variation source	Diagnosis
P1	*KCNE1*	c.246dupC	Likely pathogenic	M	LQT2
		(p.Glu83Argfs*27)			
P2	*KCNH2*	c.1789T>G	Likely pathogenic	M	LQT5
		(p.Tyr597Asp)			
P3	*KCNJ2*	c.224C>T	Likely pathogenic	M	LQT7
		(p.Thr75Met)			
P4	*KCNH2*	c.851G>T	Uncertain	M	LQT2
		(p.Ser284Ile)			
P5/P6	*KCNQ1*	c.905C>T	Pathogenic	M & F	LQT1
		(p.Ala302Val)			
		c.674C>T	Likely pathogenic		
		(p.Ser225Leu)			
P7	*KCNQ1*	c.905C>T	Pathogenic	M & F	LQT1
		(p.Ala302Val)			
		c.552C>G	Likely pathogenic		
		(p.Tyr184Ter)			
P8	*CACNA1C*	c.4714del	Likely pathogenic	F	LQT8
		(p.Ala1572ProfsTer15)			
		c.1216G>A	Pathogenic	Spontaneous	
		(p.Gly406Arg)			
P9	*KCNQ1*	c.1702G>A	Pathogenic	M	LQT1
		(p.Gly568Arg)			
P10	*KCNQ1*	c.569G>A	Pathogenic	F	LQT1
		(p.Arg190Gln)			
P11	*KCNH2*	c.1682C>T	Pathogenic	F	LQT2
		(p.Ala561Val)			
P12	*KCNH2*	c.3407_3456del	Uncertain	M	LQT2
		(p.Leu1136ProfsTer117)			
P13/P14	*KCNQ1*	c.1486_1487delCT	Pathogenic	–	LQT1
		(p.Leu496Alafs*19)			
P15	None	–	–	–	LQTS

“M” means “mother”, “F” means “father”.

## 4 Discussion

The functional gain or loss of various cardiac ion channels and regulatory proteins can lead to LQTS (6, 21). Among these, the pathogenicity of mutations in *KCNQ1*, *KCNH2*, and *SCN5A* has been well-established: Specifically, *KCNQ1* and *KCNH2* encode delayed rectifier potassium channels, and their mutations lead to a loss-of-function, affecting I_Ks_ and I_Kr_ during the plateau phase of the myocardial action potential. Conversely, *SCN5A* encodes a voltage-gated sodium channel and its mutation results in a gain-of-function that primarily impacts I_Na_ during the rapid depolarization phase (22–24). Eventually, any abnormality of these currents causes delayed repolarization in cardiomyocytes. Some studies have reported that more than 80% of LQTS patients with genetic abnormalities harbor mutations in *KCNQ1* and *KCNH2* (2, 25). Among the children in our study, *KCNQ1* and *KCNH2* exhibited detection rate at 73.3% (11/15), consistent with the reported results.

**Figure 2:**
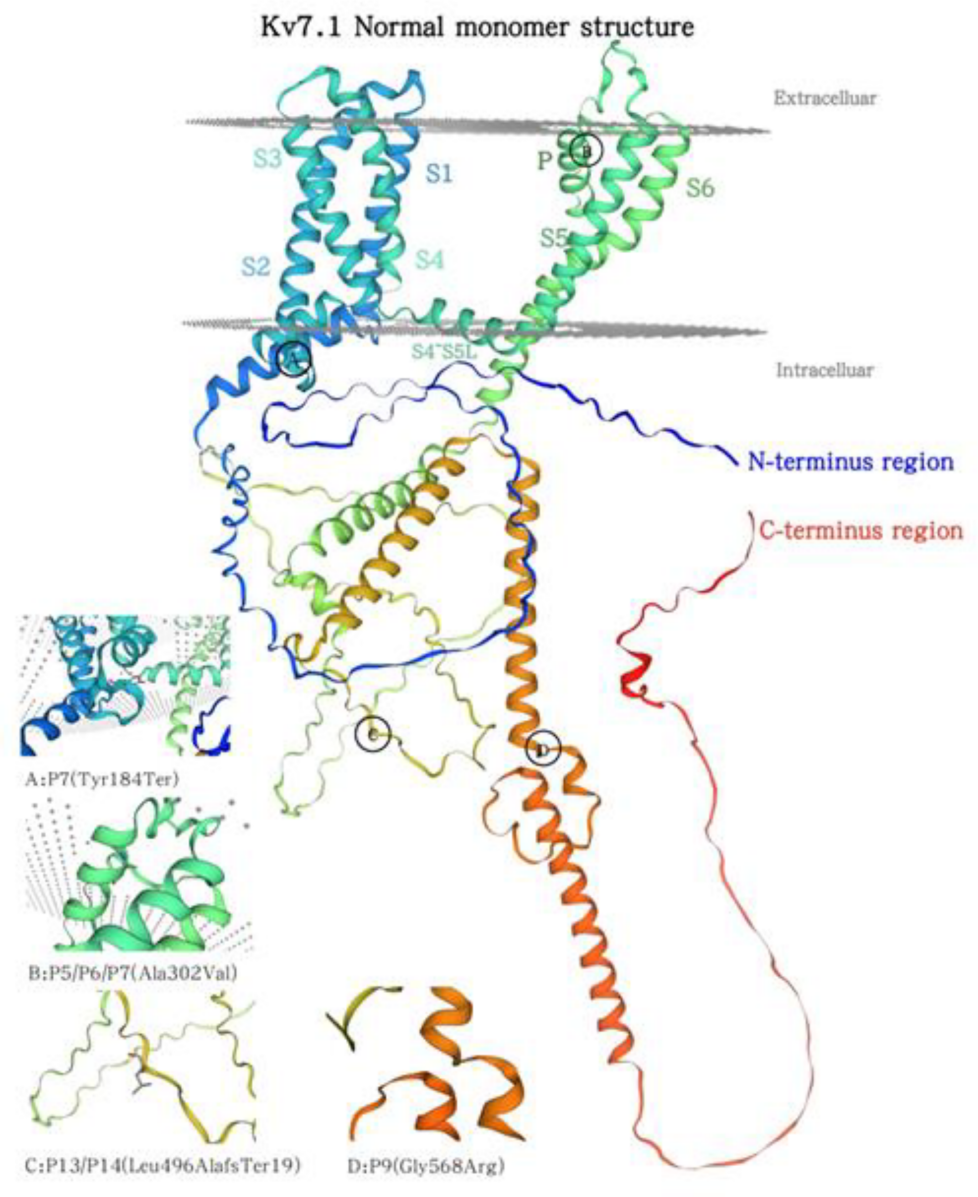
Normal Kv7.1 monomer structure alongside aforementioned family mutation sites.

The predominant mutation type found in our study was missense mutations (73.3%, 11/15). *KCNQ1* encodes the *α*-subunit of a potassium channel, with each domain consisting of six transmembrane segments and an interdomain connector. The S5-Pore-S6 serves as the pore-forming region, a critical component of the Kv7.1 channel (2). Studies indicate that amino acid alterations in both S5-Pore-S6 and C-terminal region correlate with an increased risk of adverse cardiac events (26, 27). Among the five *KCNQ1* mutation families in this study, two families harbored missense mutations (P5/P6 and P9) within these regions; one family (P7) exhibited both missense and nonsense mutations affecting this region. Additionally, another family (P13/P14) had a large-fragment frameshift mutation in the S5-P-S6 region, leading to premature termination of translation and partial deletion of the C-terminal region—a mechanism that may explain its pathogenesis. Figure 2 illustrates the wild-type Kv7.1 monomer structure and the mutation site identified in this study (Figure 2).

Upon comparison with ClinVar and Uniprot databases, seven previously unreported mutations were identified among this cohort: *KCNE1(c.246dupC)*, *KCNH2(c.1789T>G, c.851G>T, c.3407_3456del)*, *KCNQ1(c.552C>G, c.1486_1487delCT)*, *CACNA1C(c.4714del)*. Cross-referencing multiple genetic databases revealed variations at specific mutation sites, where alterations in nucleotide composition or corresponding amino acid sequences were documented. The *KCNH2 (c.1789T>G)* occurs within a critical helical structure (residues 583–597) positioned at the channel's pore entrance, serving as a bridge to voltage-sensitive components. This structure suggests potential impairment of normal ion flow through the channel, likely contributing to dysfunctional electrical signaling (28–30). Regarding the *KCNE1 (c.246dupC)* and *(KCNH2 c.3407_3456del)*, while their exact pathophysiological roles remain undefined, grounded on the frameshift mutations could prematurely halt protein synthesis. This would likely produce truncated channel proteins missing essential structural elements, potentially disrupting their three-dimensional architecture. Experimental validation through genetically modified animal models may help confirm the clinical significance of these genetic changes and their relationship to observed cardiac abnormalities.

*KCNH2 (c.851G>T)* variant causes a substitution of amino acid 284 from serine to isoleucine in the protein's primary structure. Some studies suggest that splicing alterations at this site do not significantly impair the functional properties of Kv11.1 (31), and it is considered to be “uncertain pathogenic” according to the ACMG guidelines. However, in this patient (P4), syncope was present, the QTc was 558 ms and Schwartz score was 4, which resulted in clinical diagnosis of LQTS excluding other secondary factors. This discrepancy implies that *in vitro* functional predictions may not fully reflect *in vivo* protein behavior.

*CACNA1C (c.4714del)* variant induces a frameshift mutation at nucleotide 1,216, transitioning from guanine to adenine, which leads to premature termination of translation. Nevertheless, there is currently no literature reporting its functional implications, so it has been classified as “likely pathogenic” in accordance with the ACMG guidelines. To ascertain whether this site constitutes a pathogenic mutation, further investigations are warranted.

Additionally, another patient (P15) with a Schwartz score of 4 could be clinically diagnosed as LQTS after excluding other secondary factors while no related abnormalities were identified in the genetic test results. Current research indicates that the genetic screening yield for LQTS demonstrates suboptimal diagnostic sensitivity, with existing literature reporting molecular detection rates approximating 70%–89% in clinical cohorts (18, 19). However, some studies have demonstrated that LQTS may arise from mutations within the intron regions in certain patients (32, 33). This highlights the limitations of our study given that the genetic testing conducted in our group only encompasses the exon regions along with the upper and lower 20 bp of introns.

Extramiana F. et al. observed no gender difference in QTc in LQT1 patients under 15 years of age in a retrospective study (34). However, in our cohort, female children exhibited significantly longer QTc values (mean ± SD: 498 ± 32 ms vs. 462 ± 28 ms; *P* = 0.008), a discrepancy that may stem from small sample size or differences in age stratification criteria. Ozawa J. et al. found that among LQT2 children older than 13 years, female children had a higher risk of adverse cardiac events (35). Some studies suggests that estradiol has a role in delaying cardiac repolarization (36, 37), so it is supposed that gender differences in QTc were amplify after puberty, but the specific mechanism has not been elucidated thoroughly. Additionally, Ram K. Rohatgi et al. found that in children with LQTS, those with longer mean and maximum QTc were more likely to have adverse cardiac events, QTc was proposed as a risk prediction (38).

Emotional stress or fatigue appear more common triggers in pediatric genetic heart disease patients than in adults. Based on current reviews and guidelines, some patients with LQTS may experience cardiac adverse events due to certain triggers: exercise-induced events in LQT, auditory stimuli in LQT2, and rest/sleep-associated events in LQT3, whereas emotional stress is implicated both LQT1 and LQT2 (2–4, 39). This investigation revealed strenuous exercise/emotional stress was the primary inducement in LQT1 (7/7), and emotional stimulation predominant in LQT2 (3/4), which is consistent with the reports above. Notably, one LQT1 patient reported after exposure to fatigue and high-temperature environments mirroring a fatigue-induced LQT11 case described by Li et al. (40), too. These observations underscore the diverse triggers of LQTS-related cardiac events, emphasizing the need for comprehensive clinical evaluation.

In this cohort, 73.7% of pediatric patients manifested clinical features of syncope, seizures, or cardiorespiratory arrest. Although current guidelines identify syncope and cardiorespiratory arrest as primary presentations, prior studies report lower incidence rates (25%– 28.3%) in general LQTS populations (16, 41). This discrepancy may be attributable to the selective inclusion of pediatric cases who underwent systematic genetic evaluation, with the cohort demonstrating a substantial diagnostic yield of pathogenic variants (14/15 cases). Notably, two LQT2 patients (P4, P12) exhibited seizure-predominant phenotypes and purported “epilepsy” family histories, though epileptiform activity was excluded following comprehensive electroencephalogram evaluation. Previous research indicates a portion of LQT2 patients may present seizure-like manifestations (42). These observations underscore the necessity to consider LQT2 in children presenting with prolonged QTc intervals and seizure activity, with EEG monitoring recommended for differential diagnosis when clinically indicated.

Emerging evidence indicates the pathogenicity of compound mutations in channelopathy progression. Gaku Izumi et al. demonstrated significantly elevated risks of syncope and mortality in patients harboring multigenic variants compared to monogenic carriers (43). This finding aligns with Hideki Itoh et al.'s observation of prolonged QTc intervals and earlier onset of major cardiac events in compound mutation patients (44). Our cohort analysis identified four pediatric cases with biallelic pathogenic variants, three-quarters of whom manifested life-threatening arrhythmic events (2 syncope, 1 ACA), demonstrating a potential genotype-phenotype correlation. These collective findings suggest multigenic interactions may amplify electrophysiological instability through synergistic ion channel dysfunction. The intricate genotype-phenotype correlations in channelopathies remain incompletely elucidated, necessitating systematic exploration of variant pathogenicity.

However, our study does have certain limitations. Firstly, LQTS is a rare condition with a low incidence in the pediatric population, and a notable limitation of this study is the small number of cases included. Nonetheless, the findings align with those of other reported studies. LQT1 and LQT2 are identified as the most common types (80%), while LQT5, LQT7, and LQT8 are observed as rarer variants. Further investigation with a larger sample size may be necessary to determine whether there is greater variability in LQT types in Southwest China. Secondly, LQTS is a genetic disorder, various studies have indicated that the gene detection positive rate is not 100%. Some research reports suggest this rate is approximately 70%. The potential oversight of intronic mutations in pediatric LQTS patients, combined with the disease's marked phenotypic and genotypic complexity, suggests that future implementation of whole-genome sequencing may enable more comprehensive detection of pathogenic variants across genomic regions.

In conclusion, although LQTS is predominantly attributed to genetic mutations, the pathogenic mechanism of associated genes and the etiology of cases without identified mutations remain to be fully elucidated. The QTc of electrocardiogram and main symptoms (e.g., syncope, arrhythmias, ACA) serve as key diagnostic markers. A comprehensive evaluation—including thorough investigation of family history and application of the Schwartz scoring system—is essential. For patients with atypical presentations, genetic testing provides critical diagnostic support, particularly in identifying pathogenic variants that may guide risk stratification and therapeutic decisions.

## Data Availability

The original contributions presented in the study are publicly available. This Protein sequence can be found here: [Uniprot accession number: P51787-1] (https://www.uniprot.org/uniprotkb/P51787/entry#sequences).
